# Analysis of the retention of women in higher education STEM programs

**DOI:** 10.1057/s41599-023-01588-z

**Published:** 2023-03-11

**Authors:** Gabriela Ortiz-Martínez, Patricia Vázquez-Villegas, María Ileana Ruiz-Cantisani, Mónica Delgado-Fabián, Danna A. Conejo-Márquez, Jorge Membrillo-Hernández

**Affiliations:** 1 School of Engineering and Sciences, Tecnologico de Monterrey, Monterrey, Mexico; 2 Institute for the Future of Education, Tecnologico de Monterrey, Monterrey, Mexico

**Keywords:** Education, Cultural and media studies

## Abstract

Gender equity and quality education are Sustainable Development Goals that are present when a culture of equity and inclusion is pursued in society, companies, and institutions. Particularly in undergraduate programs in Science, Technology, Engineering, and Mathematics (STEM), there is a noticeable gender gap between men and women. The objective of this study was to find out the causes of permanence in STEM careers of women, as well as the possible causes of career abandonment towards another STEM or non-STEM career. This was done by analyzing historical data for admission to STEM careers and using an instrument (survey) for data collection carried out in a private university in Mexico. Historical data indicates that only 17% of the total population were women choosing a STEM career. A survey was carried out for 3 months to obtain information on the factors that affect the decision to opt for a STEM career or to remain in it. It was found that men and women prefer inspiring Faculty who motivate them to continue their careers. Factors such as the competitive environment and the difficulty of teaching with less empathetic Faculty were negative and decisive aspects of decision-making. School achievement did not influence the dropout rate of women in STEM careers. The factors of choice and desertion of women in STEM careers were determined, and actions of educational innovation such as mentoring and timely monitoring of already enrolled female students, digital platforms for students and Faculty, awareness workshops for Faculty, and talks with successful women in STEM areas were proposed.

## Introduction

Science, Technology, Engineering, and Mathematics (STEM) careers are classified into two groups: “applied” science (computer science, engineering, and engineering technologies) and “pure” science (biology, chemistry, physics, environmental science, mathematics, and statistics) (Deming and Noray, 2018). They are often perceived as too complex, require too much education, and are taught by specialized instructors (Kier et al., 2014). Given their biological scientific content and the concurrent interest in medical sciences, the medicine and health area has also been included in STEM studies (Bennett et al., 2021; Heo et al., 2022). Usually, male students have reported more interest in physical sciences and females in the biological sciences (Kier et al., 2014).

Despite efforts to achieve gender equity in all aspects of society, there is still a gender gap in the number of professionals graduating from STEM careers: graduated women do not exceed 30%. According to the UNESCO Institute for Statistics, the mean percentage of female students in tertiary education enrolled in engineering, manufacturing, and construction programs was between 6% and 7% between 2015 and 2018; in contrast, the percentage of male students choosing these careers is around 20–21%. This gap shows differences between countries and is even more significant in countries with biases due to gender stereotypes or cultural norms that influence female behavior and the family environment of girls and women. Unfortunately, women today have more to consider than simply doing what they love. Such limiting considerations, instilled in them from an early age, could be salary, work environment, and social stereotypes about what they can do, even today in some countries, there are even “activities for women” and “professions for women”, “professions for men”, “jobs only for women” or “jobs only for men” (Camacho et al., 2021).

Organizations across the world are working on reducing the gender gap in STEM. Still, the situation depends on many factors related to not only cultural and socio-economic context but also factors such as self-perception, self-efficacy, or previous educational experiences (Cadaret et al., 2017; Leaper and Starr, 2019; Lent et al., 1994, 2002; Moss-Racusin et al., 2012; Malik and Al-Emran, 2018; Salami, 2007; Salas-Morera et al., 2019; Seo et al., 2017). In addition, throughout the academic and professional trajectory of a woman in the STEM area, there are many stages where the number of women decreases: when they enter university, when they enter the labor market, and when they reach high professional positions (Amon, 2017; Seo et al., 2017).

Historical examples of excellence and leadership in STEM areas, such as Marie Curie, Barbara McClintock, Rosalind Franklin, and Marissa Mayer, have been paramount in serving as role models for students who want to pursue a STEM career. However, the fact that they are the exception and not the rule indicates a latent problem. Additionally, there currently needs to be more recognition of women in the workplace related to STEM careers, discouraging students from pursuing a career in these areas. Therefore, it is essential to reflect on strategies to (1) encourage women to choose STEM careers, (2) maintain enrollment already enrolled in STEM careers to avoid dropouts, and (3) achieve gender parity in higher education institutions (HEIs) in all areas and levels (managers, Faculty, and collaborators).

## Literature review

**Fig. 1:**
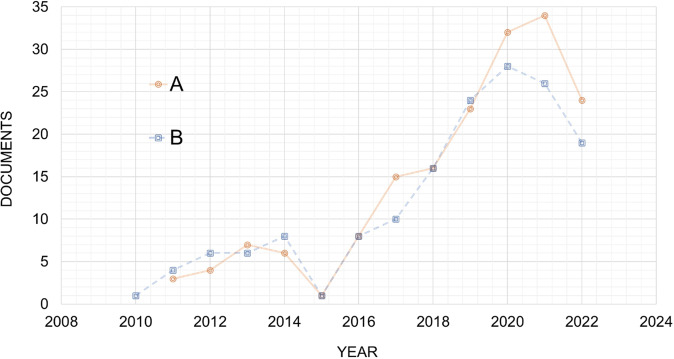
The number of documents by year obtained from the SCOPUS database using search strings. **A** “STEM career” AND “gender” AND “dropout” (173 hits) and **B** “STEM career” AND “women” AND “dropout’ (157 hits) in all document fields.

According to a search in the Scopus database (Fig. 1), this research field started 10 years ago, and since the COVID-19 pandemic, the number of research studies on this topic still needs to be higher. Although this fact is well known, studies about specific women’s reasons for leaving STEM careers and the success of efforts to avoid such behavior have not been extensive. Among specific assumptions explaining why female students drop out of STEM careers, the theory of marginalization and validation apply, especially since they are more likely to doubt their abilities or give in to stereotypes (Louten, 2022). In the United States, women are less persistent than men in completing a STEM degree (48% vs. 65%) because they consider themselves to have a low level of self-efficacy, despite being equally prepared (Koch et al., 2022). Studies confirm that the problem lies in social factors (such as gender equity and life satisfaction) and the value women give to studying one career compared to another (Stoet and Geary, 2018). Women do not even consider themselves capable of pursuing a STEM career (Cuevas et al., 2022). Family, school, and Faculty are unsupportive (Tandrayen-Ragoobur and Gokulsing, 2021). Some researchers have remarked that women have more social interests than men, who are more interested in working with things; thus, if social orientation were emphasized in STEM careers, more women would be in those areas (Salzman and Lieff, 2019; Struyf et al., 2017; Tyler-Wood et al., 2018). Specific studies to investigate exactly which part of the STEM careers they decide to leave and the reasons behind that decision are needed to propose interventions that can promote a change (Vooren et al., 2022). In this regard, Louten (2022) implemented a program with a series of components based on the literature that increased the retention of women in STEM careers, including psychosocial adjustment, support for challenges, educational activities and proposals, milestones of the academic trajectory, and achievement of objectives. Another strategy has been to make Faculty aware of the gender difference when students decide to leave the STEM career since they are not aware of the phenomenon or the motivations that this entails, to generate strategies that reduce sexist behaviors and stereotypes (Cavaco et al., 2021; Isphording and Qendrai, 2019; Wee and Yap, 2021).

The Organization for Economic Co-operation and Development indicates that for every 10 men, only three women will select a STEM career focusing on contributions to science and technology, widening the gender gap. An important aspect to consider is the reality of HEIs in regions such as Latin America, where cultural elements and norms of conduct towards women have a decisive influence on their selection and permanence in a STEM career. To reduce the dropout rate of women in STEM careers, our university developed a pilot program between April and June 2019. This program consisted of mentoring and monitoring female students in STEM areas. In this program (Women in Science and Engineering), knowledge and skills were shared through small groups, helping others in their lives and careers. Fifty-five students from 25 different institutions in 3 countries were served, with 27 instructors as mentors. The main objective of this program was the monitoring and identification of the reasons why students do not consider entering STEM areas. This pilot work was the detonator of the present investigation when trying to identify the causes of the desertion of female students in STEM areas.

In the United Nations 2030 Agenda, Sustainable Development Goals (SDGs) 4 and 5 are relevant to our educational work. One of the objectives established to promote a more sustainable world is gender parity, as established in SDG5: “Gender equality is not only a fundamental human right but a necessary foundation for a peaceful world, prosperous and sustainable” (United Nations, 2019). Gender balance in STEM is part of this challenge, and it has a double dimension: vertical and horizontal (Fulcher and Coyle, 2011). Scientists and politicians developed many different frameworks regarding vertical balance to study what influences the career in STEM and how to support a balanced environment in academia (Bührer et al., 2019; Wolffram et al., 2017) and in the industry (Beede et al., 2011; González-González et al., 2018; Lambrecht and Tucker, 2019; Sassler et al., 2017). A large-scale longitudinal study in the USA found that after 12 years from graduation, around 50% of women had left their job in the STEM field. Comparing these figures with the general one, the work shift cannot be linked to family factors. In contrast, the work environment and the related job characteristics emerged as the key features (Glass et al., 2013). However, family factors also play a role in the recruitment stage because STEM careers are perceived to fight with family goals (Weisgram and Diekman, 2015). This explains why having a female family member in the STEM field favors girls’ STEM interests (Cowgill et al., 2021). Looking at the horizontal segregation, it is essential to distinguish recruitment from the retention campaign. The first refers to reinforcement of the attraction of young girls in the field, while the second aims to increase the retention of those already enrolled in STEM and support their entrance into the labor market. Those are typically addressed with various strategies, and it is easy to mix the two campaigns. Steele and Aronson (1995) highlighted the importance of “rendering the right students the right intervention”. For example, role models are valuable and effective in both moments. However, for the recruitment phase, it is important to have female role models (Makarova et al., 2019). For retention, one can have male and female role models to reach the desired goal (European Union, 2018). In recruitment, the women’s intrinsic and extrinsic motivations concerning their academic decision have a dominant role. At the same time, in retention, the rationale mostly comes from personal stimuli and experiences. A review of the instruments used to study the gender gap in STEM stated that the main variables in these motivations are the influence established by this decision and the education path, the related achievements, the recommendations and work of parents, the stereotyped ideas they have towards this sector (Verdugo-Castro et al., 2019). Another factor that one must consider is the attitude toward STEM, which can be measured by spatial ability and by the mental rotation factor, as shown in a longitudinal study of over 50 years (Wai et al., 2009).

How to translate all this input into a coherent and appropriately effective campaign? Many institutions have tried to answer this question through case studies (García-Holgado et al., 2019; Politecnico di Torino, 2019), projects (Ballatore et al., 2020; García-Holgado et al., 2020a), and events (Wyred, 2019). Currently, attraction campaigns use different media to foster a more balanced field, from the more traditionalist type (i.e., conferences, speech) to more interactive ones (i.e., summer schools, hackathons). Recently technology has been used to spread the message among the youngest easily (García-Holgado et al., 2020b). Although the majority tend to forget the importance of increasing the retention of enrolled women, only a few experiences have been scientifically analyzed (Gómez-Soler et al., 2020). In general, the emphasis placed directly on the issue of the gender gap during the mediated strategy and events appears relevant. It is essential to make women feel welcome and not to emphasize the vertical dimension of the gender gap (ceiling effect, salary mismatch, and so on) in order not to have the opposite effect (Drury et al., 2011).

Women are persistently underrepresented in STEM (UNESCO, 2007; OECD, 2015; Tomassini, 2021; UNESCO Institute for Statistics, 2018; European Commission, 2021). The problem of the low number of women in STEM careers has been previously reported. For example, Walton and Spencer (2009) listed some factors that prevent women from choosing STEM careers, the school system, grades, and social prejudices. Specifically, they suggested that evaluation has something to do with stereotypes and prejudices rather than individual abilities or potential. Although most countries have more women than men enrolled in tertiary education, the number of women in tertiary education who choose STEM is around 15%. For example, only 13.76% of women in tertiary education choose STEM compared to 35.12% of men in Colombia [0.39 gender gap score (35.12/13.76)]. The situation is worst in Spain, with a gender gap score of 0.33, Finland with 0.25, and Ireland with 0.38.

Additionally, female STEM scholars experience one of the highest rates of sexual harassment in any profession by faculty or staff (National Academies of Sciences, Engineering, and Medicine, 2018). It is well known that the lack of women in STEM is therefore not only affected by socio-cultural factors but also by the bullying they experience in these professions, the lack of participation during elementary, middle, and high school, and the lack of idols that inspire more women to take their place in these careers and continue to be essential for their improvement (Rosales-Rodríguez, 2020). Even though female STEM graduates have better job outcomes (Moso-Diez et al., 2021) and despite narrowing the gender gap, women continue to be underrepresented (Huang et al., 2020). This gap is also due to a lack of motivation. They are affected by socio-cultural factors, gender-based preconceptions and biases, field-specific skill beliefs, lifestyle values, work-family balance preferences, and inclinations or professional desires (Alam, 2022). In recent decades there have been relatively significant improvements in advancing gender equality in various dimensions of human life. This gap depicts differences between countries and is highlighted in countries that show biases due to gender stereotypes or cultural norms that influence female behavior and the family environment of girls and women. We can find that sociocultural factors, even though they have made it possible to improve women’s living conditions, continue to limit them when choosing their careers. Women must consider more issues than simply doing what they like. Such limiting considerations, instilled in them from a young age, could be salary, work environment, and social stereotypes about what they can do (Camacho et al., 2021). Recently, García-Peñalvo et al. (2022) published a book on Women in STEM in Higher Education (HE), where they discuss some issues about why there is this disparity between men and women in STEM careers relevant to this article. The chapter by Campos et al. (2022) maps the most cited reports on the retention of women in STEM careers from 2011 to 2021.

### The Social Cognitive Career Theory (SCCT)

Part of the decision of the career to be studied (STEM or not) and the prevention of dropouts have to do with the Social Cognitive Career Theory (SCCT), which aims to explain three aspects of professional development: (1) how to develop academic and career interests (2) how educational and career decisions are made, and (3) how academic and career success is achieved. The theory incorporates interests, abilities, values, and environmental factors affecting professional development. Established by Lent et al. (1994), the SCCT is based on Albert Bandura’s general social cognitive theory (Bandura, 1965, 1971), a seminal theory of cognitive and motivational processes that has been extended to the study of many areas of psychosocial functioning, such as academic performance, health behavior, and organizational development. This theory involves three fundamental components: *self-efficacy beliefs*, *expectations of results*, and *objectives*. Which, of course, are part of what female students in STEM careers experience. *Self-efficacy* refers to an individual’s confidence in their abilities to perform an assigned task. The SCCT assumes that people are likely to be interested in, choose to perform specific tasks, and perform better in activities in which they have solid self-efficacy beliefs, provided they also have the necessary skills and environmental support to perform these activities. For this reason, for women in STEM careers, the vocational orientation for career choice, tutoring, and academic and professional follow-up is fundamental. *Expectations of results* refer to expectations about the consequences or results of performing a task, a course, or achieving a particular goal. Students choose a career because by having an academic degree, they will be able to develop professionally. Therefore, the work environment is carefully analyzed before choosing a career, which is why this element is crucial to encourage women to choose a STEM career. Personal goals are divided into two *objectives* according to the SCCT: *selection* and *performance objectives*. By setting goals, individuals organize and guide their behavior. The social cognitive theory posits that goals are significantly related to both self-efficacy and outcome expectations: people tend to set goals consistent with their views of their abilities and the results they hope to achieve by pursuing a particular course of action. A woman’s choice of a STEM career is affected by these three components, and success or failure in achieving personal goals, in turn, becomes essential information that helps modify or confirm self-efficacy beliefs and expectations of results.

## Study objectives

The problem of the gender gap in STEM careers in HEIs is a stigma that has accompanied professional development. Thus, there is an express need to understand the causes based or not on the SCCT theory and determine, based on a systematic study with today’s students (post COVID-19 pandemic and generation Z), the current causes and, based on those, propose actions that HEIs could consider reducing the gender gap, which would result in a more fair and equitable society. Thus, two research questions are intended to be taken as a basis for this study:

1. What are the internal and external factors affecting the permanence of female students in STEM programs at a private multi-campus university in Mexico? How is this related to the SCCT theory?
2. Based on the results of research question 1, what actions would favor the retention of women already enrolled in STEM career study programs?

First, a diagnosis of the current state of STEM programs was essential to validate if there was a significant gap in women studying STEM careers at the Tecnologico de Monterrey, the fifth-best university in Latin America and the best private university in Mexico, according to the QS 2022 Ranking. The number of women entering STEM careers was determined based on the data available in the historical archives. Afterward, we applied a survey to students studying different semesters in academic STEM programs. Based on the results, we propose long-term goals that can increase the number of women entering STEM educational programs.

## Methodology

This work was carried out in three stages:

- Diagnosis of the current state of STEM programs considering the number of women enrolled in undergraduate STEM academic programs.
- Application of a survey to students enrolled in undergraduate STEM academic programs.
- Analysis of the data collected and proposals for action to reduce the existing gender gap.

### Diagnosis of the current state of STEM programs

A detailed database of all students was built (University Data Hub; UDH). This circumstance allowed us to carry out a detailed study of the students enrolled in STEM careers. The available data was from the fall semester of 2014. The UDH safeguards the enrollment, transfer, dropout, and completion data of all students from the 26 campuses nationwide of Tecnologico de Monterrey. The always-protected personal data provided by the UDH were handled anonymously. Data from the School of Sciences and Engineering and the School of Medicine and Health Sciences were analyzed concerning (i) the percentage of enrollment of female students in academic engineering and science programs, (ii) the percentage of retention of female students in engineering and science academic programs, and (iii) historical data on academic performance. The data on those schools were selected because of data availability. Although some careers in Built Environment and Creative Studies areas, such as Architecture, Civil Engineering, Bachelor of Music Technology and Production, or Digital Arts, also contain many subjects on technology, these were not considered in this study owing to the difficulties of working with the data interface.

### Application of a survey to students of STEM programs

A survey instrument was developed to identify (i) the leading factors related to the retention of students in undergraduate STEM academic programs and (ii) define the components of the sense of community and cognitive career decision-making, based on the SCCT (Lent et al., 2002), based on interests, choice, and success/performance in STEM areas. The instrument served to define the external and internal factors that would have influenced the decision of women to choose a STEM or non-STEM career. Table 1 shows an example of the instrument designed. The survey was designed to obtain demographic information (year of college entry, gender, and campus), career options, whether students changed majors during their studies, and how they identify with their current careers. No personal data was collected. Other questions aimed to learn about their past educational experiences (i.e., high school courses or extra-curricular experiences in STEM). Finally, open-ended questions asked them to write about their perception of four factors that may have affected their career selection, whether they are in the same area or have switched to another. These factors are the attitudes of others, one’s attitudes and values, external situations, and the actions of Faculty and classmates. The applied survey can be downloaded from the supplementary material. The information obtained from participants was anonymized. By filling out the questionnaire, students accepted that the authors could use any information to conduct research and other related activities. The survey was implemented under a random pop-up sample design, made as a snowball or chain (Leighton et al., 2021). The responses from March to June 2022 were taken for study.

**Table 1:** Exercise for survey design, based on the SCCT theory.

Type	Factor	Examples/reasons	General question
External	Attitudes toward minorities	Social attitudes toward sexual minorities, gender prejudice, gender stereotypes	What attitudes have impacted you in staying or not in your engineering studies?
	General environment situations	Hostile experiences (tend to be overlooked as isolated incidents rather than systemic problems)	What situations have impacted you in staying or not in your engineering studies?
	Situations of the academic environment	Interactive engagement with students during class, mentoring and support from faculty, tutoring programs within the university or department	What actions by Faculty and students have impacted you in staying or not in your studies?
Internal	Sense of belonging and identification with the race	Sense of belonging, identification with STEM. Identity seems to be the strongest predictor of retention	What identifies you with the program you chose? What changed?
	Beliefs, values, attitudes	A woman’s persistence in STEM has been linked to her internal motivation or commitment, beliefs, and resilience toward gender stereotypes	What attitudes or qualities have impacted your selection and permanence in the program?
	Previous academic preparation and experience	Adequate pre-college education, various learning experiences such as appraisals from previous achievements in each domain, educational backgrounds, career adaptability	Describe your training and previous experiences that you consider impacted your program selection?

### Analysis of data collection and proposals

A descriptive quantitative analysis of the survey results was performed. Open responses were assessed through qualitative data analysis using a stratified prospective analysis method to illustrate the characteristics of the subgroup of interest under the postpositivist and critical theory approaches (Laumann, 2020). Analysis of demographic data and academic performance was conducted using the UDH. On the other hand, the theory of change was used to draft proposals to increase the enrollment and retention of female students in undergraduate STEM academic programs.

## Results and discussion

### Diagnosis of the current state of STEM programs

The Tecnologico de Monterrey is the best private university in Mexico, with a presence in 26 cities. When analyzing the UDH data, it was observed that historically there had been a low number of women in STEM careers (considering the careers of the Schools of Engineering and Sciences and Health Sciences). Accordingly, during 6 years, from Fall 2014 to Fall 2020, 77,517 students (100%) were enrolled in the university; 45% were women (34,703). Of the total number of students, 49% chose a STEM major (men and women; 37,984), and of the total number of students in STEM majors, only 36% were women (13,675). This means that only 17% of the total (17,517) were women in STEM.

In 2014, Tecnologico de Monterrey gradually began implementing a new educational model, the Tec21 Model (https://tec.mx/es/tec21). This educational model, based on four fundamental pillars: (a) Challenge based learning; (b) Flexible education; (c) Inspiring Faculty; (d) Education in a memorable, comprehensive, and *inclusive environment* (Membrillo-Hernández et al., 2022), was fully implemented in 2021. One of the first actions accompanying the new educational model was the Women in Engineering and Sciences (WiS) initiative, which brings together a team of professors, collaborators, and authorities to create policies and include activities such as seminars on highly successful women in STEM careers or women CEOs of major companies and startups, workshops, mentoring programs, scholarships, and other activities to encourage women to choose and stay in STEM careers. However, only an increase of almost 2% has been observed in the number of women that chose STEM careers from the total enrolled women in the new educational model (data from 2019 and 2020). Thus, more work is needed in this regard.

**Fig. 2:**
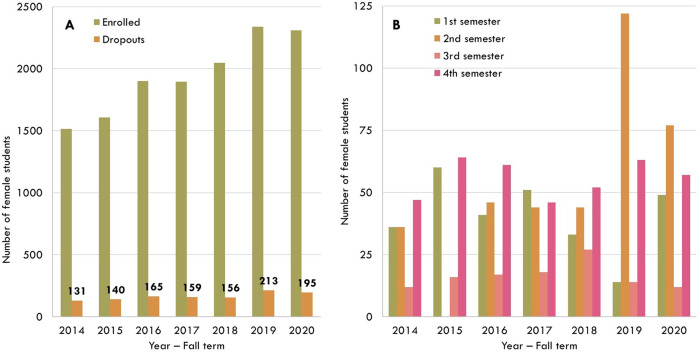
The number of female students enrolled in STEM careers from Fall 2014 to Fall 2022. **A** Students enrolled per year and dropouts (data labels) from the same generation of female students. **B** Distribution of dropouts per semester considering the total number from the **A** panel.

When dropouts were analyzed, it was evidenced that a slightly higher percentage of female students than male students dropped out of STEM careers (8.60% vs. 8.35%). Notably, 75% had a GPA of 70/100 or higher (70/100 is the minimum to pass), suggesting that academic performance might not be the reason to leave a STEM career path. The highest dropout rate from STEM careers occurs during the first four semesters of undergraduate studies. The second and fourth semesters accumulated the most significant number of people who changed careers (Fig. 2). This behavior has been observed in other works. Respondek et al. (2020) highlight a perceived academic control (PAC) linked to student autonomy, which is relatively higher in the first 6 months of college studies but decreases over time. There is a peak in dropouts in the first year of college. However, during the second year, PAC has only a small influence on student grades (Respondek et al., 2020). Dropouts during the second year had been related to other reasons, such as living away from family support (Sosu and Pheunpha, 2019). It has been theorized that dropping outs in further years are less probable during later years mainly because it can be more emotionally traumatizing (Sosu and Pheunpha, 2019). This may reflect that support resources at university may be of vital help in decreasing these behaviors.

Gender equality is a solid pillar of inclusive education in social justice and is one of the SDGs. Action is required to increase the number of women in STEM careers and reduce dropout rates. The information obtained from this analysis was essential to understand at what point of the career there are more dropouts and, thus, implement actions at those stages.

### Survey results

**Fig. 3:**
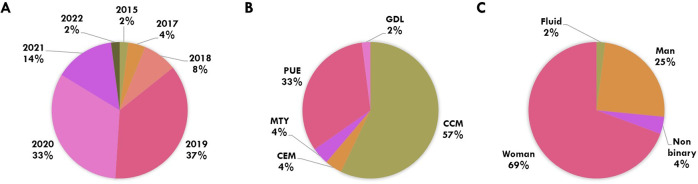
Demographic profile of survey respondents. **A** Semester (year) of admission to higher education. **B** Campus (PUE = Puebla, MTY = Monterrey, GDL = Guadalajara, CCM = Mexico City, CEM = State of Mexico). **C** Declared gender.

Considering the information from the previous section, student data collection was carried out to understand the current situation of women in STEM careers. The survey entitled “Factors that impact permanence in a STEM program” was developed on the Google Forms platform to facilitate the collection of information from students. When sharing the questionnaire with the students, the progress could have been faster, but there is no network where all the students of the STEM careers are connected. Only 49 responses (all post-COVID time) were obtained from students from different campuses. The demographic profile of the students who responded to the survey can be seen in Fig. 3. As observed, some students surveyed had between one semester and up to seven years of having started HE studies. Most respondents (78%) were close to entering or finishing their 3rd year of college by June 2022.

**Fig. 4:**
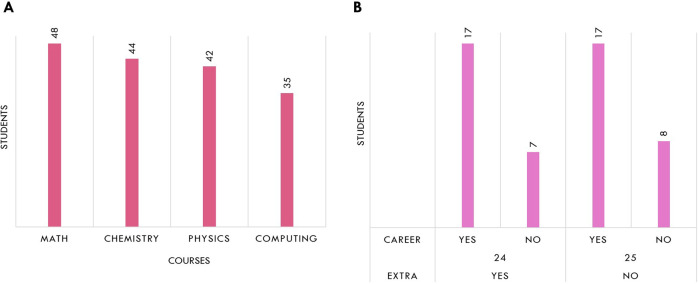
Effect of early contact (during high school) with STEM topics and its influence on the decision to study a STEM career. **A** Do you remember taking these compulsory subjects during high school? **B** If you are in a STEM career, did you take any of the extracurricular STEM courses?

One of the first questions on the survey was about STEM courses taken during high school. All the students had studied Mathematics, Physics, Chemistry, and Computing (Fig. 4A). In addition to these required subjects, some students took extracurricular STEM courses for their (non-required) interests during high school. Half of the students interviewed who are currently in STEM majors took this type of course (Fig. 4B). It is evident that prior exposure of female students to STEM-themed extracurricular activities, such as those listed in Table 2, during high school positively affects the decision to pursue a STEM career. The students who are studying STEM careers were specific when mentioning the experiences and projects they developed:

- Youth Workshop for Science of the Mexican Institute of Ecology.
- International exchange with another high school focused on engineering topics.
- Workshop for making soaps, creams, balms, shampoos, and healthy recipes based on the roselle plant (*Hibiscus sabdariffa*).
- Participation in “Chemical factor” experience with a biodegradable plastic project created from apple peels as an alternative to conventional single-use plastics to curb the concentration of microplastics in the environment and living organisms.

**Table 2:** Examples of extracurricular activities in high school mentioned by students in the survey and their STEM-related topics.

Activities	STEM topics
	Math
Labs	Biology
Fairs	Ecology
Contests	Science
HeForShe^a^	Robotics
Workshops	Chemistry
Students group	Computing
Scientific society	Programing
Beautiful patterns^b^	Engineering
	Artificial intelligence

^a^https://www.heforshe.org/es.

^b^https://dreamgrande.io/.

**Fig. 5:**
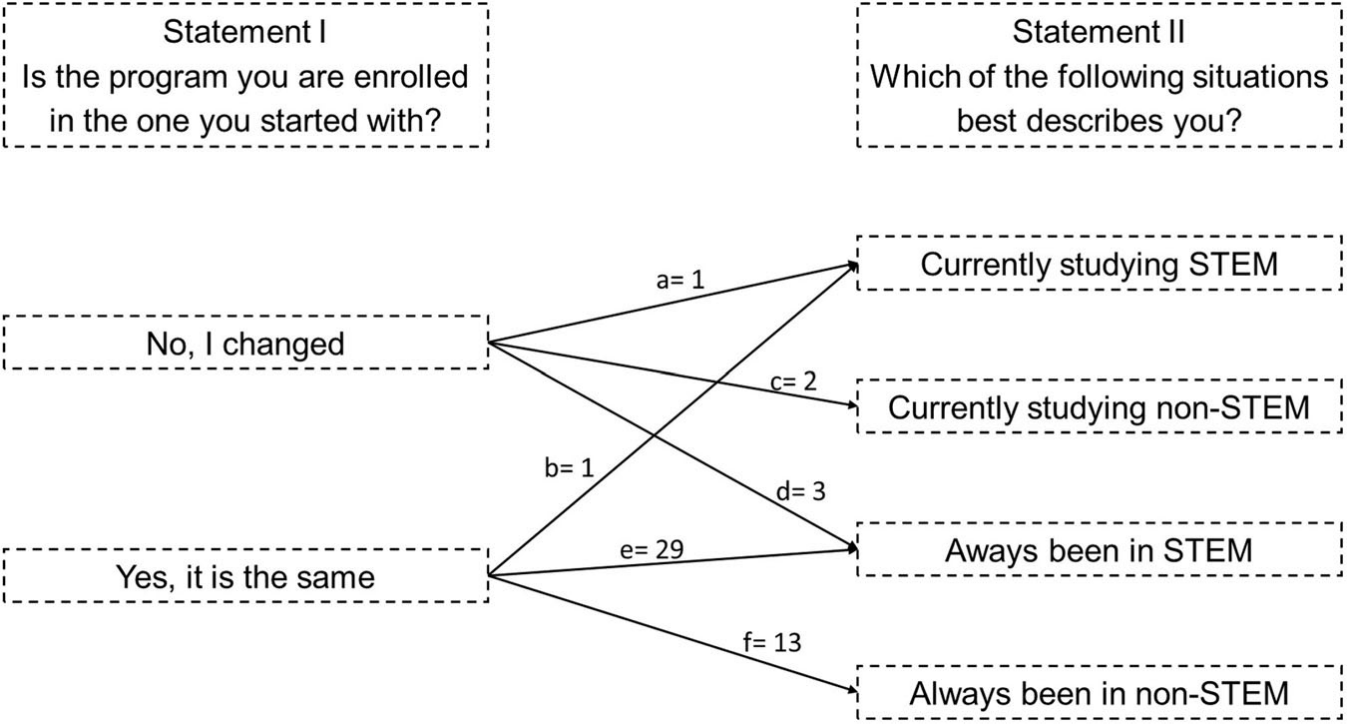
The Academic Trajectory of Students within or outside the STEM areas. According to the following statements, the students determined whether or not they started, maintained or abandoned a STEM career. Each path is identified by a letter from a to f.

Students were asked to select a few statements about their decisions after enrolling in college (Fig. 5). In some cases, the students indicated they were in a non-STEM career when they were referred to the medical field. This suggests that the development of a project involving STEM professionals before undergraduate studies arouses the intention to enter a STEM career, even when the concept of a STEM career is not 100% clear.

Among the students interviewed, those enrolled in medical courses responded that they had chosen medicine mainly because of their altruistic sense and interest in research. Those students who do not belong to STEM majors highlighted their creative, artistic, and social qualities. In contrast, those already enrolled in engineering majors stated that they love science and scientific experiments, are curious to learn, enjoy finding solutions to complex challenges, whether it is solving a scientific or a social problem, and perceive themselves as inquisitive, innovative, multidisciplinary, analytical, and critical.

The next part of the survey consisted of different questions regarding the options for Statements I and II of Fig. 5; if they changed or were always convinced and continued the same path; if they gave up studying or are not studying a STEM career, regardless of the misconceptions about the STEM career. Tables 3 through 5 summarize the topics covered in each section.

**Table 3:** Resume of attitudes, situations, and actions that impacted women’s choices towards STEM careers (*n* = 33).

Attitudes of other people	Self-attitudes and values	External situations	Actions by Faculty and students
**What […] have impacted you to stay in STEM studies?**			
Empathy, sincerity, emotion, passion for the area, accompaniment, consistency, discipline, constancy, support, and **solidarity**. Inspiring professors. Professional development and networking.	Perseverance, discipline, organization, resilience, passion, commitment, curiosity, effort, dedication, responsibility, collaboration, creativity, and inspiration. Likeness to learn and love for science.	Talking to professors about the **future**, projects with **Training Partners** or additional projects, being able to carry out laboratory practices, all applications of the career, and the subjects of the career. Self-desires and knowledge need.	Mentorship, commitment to student learning and feedback, their care, willingness to help, preparation, motivation and inspiration, support, and dedication. The atmosphere of support, friendship, and interest in having a notorious impact on the environment. The application of real situations for learning, relationship with the life and history of the human being.
**What […] have impacted you to change into STEM studies?**			
Availability and **having a common goal**.	*Tenacity, resilience and integrity, and an improvement mentality*	*To have programmed an interface* ***on my own****. Study a subject on my own and* ***understand the topics perfectly****. It made me feel, for the first time, that I was* ***capable enough***.	*Share academic means to study independently and work towards a common goal. The willingness of some instructors to give advice*.
People are very **competitive**.	*Tolerance, I´m more tolerant now*.	*There is much information that must be learned*	*All extra things that one must search for*

**Table 4:** Resume of attitudes, situations, and actions that had impacted *fluid and nonbinary gender* students in their choices toward STEM careers.

Attitudes of other people	Self-attitudes and values	External situations	Actions by Faculty and students
**A. What […] has impacted you to stay in STEM studies?**			
*Their passion for research and health sciences, interest in laboratory work, and learning more about different diseases’ pathophysiology*.	–	–	–
*Professors who have made me see that* ***science is simple***.	*My passion is to learn*.	–	–
**B. What […] have impacted you to change into non-STEM studies?**			
–	*The love for art and* ***not a comfortable life studying engineering***.	*My* ***emotional well-being***.	*The* ***competition*** *and how instructors* ***see you down***.

**Table 5:** Resume of attitudes, situations, and actions that had impacted **men’s** choices towards STEM careers.

Attitudes of other people	Self-attitudes and values	External situations	Actions by Faculty and students
**A. What […] has impacted you to stay in STEM studies?**			
Motivation, encouragement, perseverance, passion for engineering subjects, study, and knowledge. Support to help in **the exact sciences** and how interesting it seems. *The tips instructors gave me about how my career matches my* ***profile***.	Perseverance and passion for engineering subjects, likeness, interest in the topics and the career, **intelligence**, curiosity, love for science, resilience, constancy, and dedication. *That I do my best*. *I had* ***well-defined*** *what I wanted from the beginning*.	The learning is acquired through exciting projects and collaborations, salaries, experimentation, and knowledge acquisition. *My academic performance*.	Empathy, advice, good teaching, pleasure for the subjects, their support, involvement in projects with **practical applications**, they are experts in the field, experimentation, and showing interest. The mutual interest and sharing. *The different activities inspired me and gave me a perspective on what one’s future can be like as a student*.
**B. What […] has impacted you to change into STEM studies?**			
*My parents are engineers, and seeing a friend describe her love for the career*.	*Honesty, discipline, and responsibility*.	*–*	*I feel challenged and with a genuine interest*.
**B. What […] have impacted you to change into non-STEM studies?**			
*I had instructors who treated me more like a number than a person*.	*I wanted to explore my creative abilities and the opportunity to learn* ***without such extensive exams***.	*The environment was toxic: destructive competition and t****oo complicated and comprehensive tasks***.	*My instructors and classmates did* ***not empathize with*** *my difficulties*.

Regarding the context in the family environment that involves the *attitude of other people*, some women indicated that the attitudes of parents and relatives impacted them to remain in a STEM career:

- “My family has studied careers in health since my grandfather. They have always supported me to continue studying medicine, advising, and teaching me”.

- “My parents have always encouraged me to do what I love the most, no matter how challenging it is. In my house, we talk intellectually about current problems, possible implications, and solutions”.

Other women took inspiration from Faculty’s attitudes toward staying in a STEM career:

- “The feeling of empathy (when we sometimes feel tired), sincerity (about how hard a career can be or just a STEM subject, or an exam, or an exercise), excitement for the area (that they share with intensity everything what they do with us), and punctual accompaniment (learning to do things and knowing that it is okay to make mistakes, guidance, and support). At Biotechnology Engineering, the professors are very inspiring.”

- “My instructors have been a great inspiration for me, realizing that as a professional, I could develop in multiple areas, and the possibilities are great.”

However, in at least one case, a female student stated that the approach to the basics of the career with “bad instructors” pushed her to drop out and change to a different non-STEM career. The students also wrote about class or school atmosphere and their *relationships with classmates*. They referred to teamwork and the constant and motivating attitude of seeking the same goal, which is dedication and the desire to contribute and find solutions to improve our environment and the world:

- “I also like to learn from classmates with the same interests”.

- “Remind me that science is the future and that we can change the world”.

Regarding *self-attitudes and values* that had to do with their STEM career, some female students responded:

- “Resilience, honesty with myself, seeking not to compare me with my peers and understanding that I have different learning methods and topics of interest”.

- “Getting involved in activities related to my career outside of theoretical [concepts] will help me figure out what specifically I like best about my STEM academic program”.

- “The love for innovation and my desire to make technological toys”.

- “How to solve challenges with social coaches because you realize you can achieve many things”.

Some women relied on perseverance to finish their STEM studies:

- “I don’t know, knowing that I’m almost finished, although I feel that I haven’t learned much in the last semesters because the strongest is in the first semesters.”

Some other women considered that *self-efficacy* and *external situations* related to the opportunities and connections (networking) they have made during their careers had impacted them:

- “Satisfaction from completing challenges that seemed too much at first.”

- “Finishing a subject/block and seeing what is going well for me motivates me for what follows”.

- “Doing a project helped me realize that I can and do not depend on others to get good grades and valuable learning”.

- “I remember that in the 5th semester, I had doubts about whether or not to stay in the degree because the workload was overwhelming me; however, I was lucky to take the subject of plant tissue culture, where I loved working with plants and create experimental designs that were effective for solutions”.

- “One of them is to meet people who work in the profession, students and graduates [and professors], to see how they perform in different areas and the passion they feel for their work; they have inspired me a lot to keep trying and learn a lot every day”.

- “In practical terms, I have been able to help a few people within my abilities as a student. People thanked me for my help and motivated me to continue this career”.

- “I learned the value of serving during the funeral of my grandfather (who was a general practitioner), there I realized the appreciation and affection that all his patients had for him; telling stories of all the times he helped them motivated me to decide for this [career] STEM path”.

- “An important value is the actuality of a problem; constantly reading epidemiology and health statistics tells you that one of the critical areas in which more attention should be paid is health, which motivates you to be in the solution as soon as possible”.

Regarding the *actions of Faculty and students* that have impacted the students’ permanence in STEM careers, some Faculty members have had a very negative impact, as one student said:

- “Some [instructors] don’t feel like teaching or don’t know how to do it”.

However, most of the responses were positive:

- “Each instructor inspires you differently; however, in all of them, you can see their achievements and willingness to support, collaborate, and teach you. Several [former students] have also motivated me with their success stories”.

- “When I hear between the lines your reasons for continuing in science”.

- “When they talk about their work or show me what they have done, this encourages us to continue learning”.

- “The practices, mainly the accompaniment of Faculty in the pandemic [lockout], had a significant effect on us, and they were very empathetic and understanding without neglecting the seriousness of the issue”.

- “Their passion and dedication to the classes. When they talk about the things and research they do or have done. Their outstanding career and how they got to where they are now”.

- “Talk to them about the future and their advice. They are an essential part of the motivation to stay in science.”

There were few comments on the pedagogical part or teaching systems. However, the comments focused on the inspiration (repeated nine times) provided by experienced professionals since they served as role models through their passion for the subjects of the career. The students’ responses repeated the word “ future “ six times. Only one male student wrote “salary” as a factor in choosing a STEM major. Comments on reasons for not selecting a STEM major included: a sense of fierce competition among students, labeled as a toxic environment that harms emotional well-being, depersonalization of students by some Faculty who are unsympathetic or labeled as “bad” since, apparently, they do not teach correctly.

**Fig. 6:**
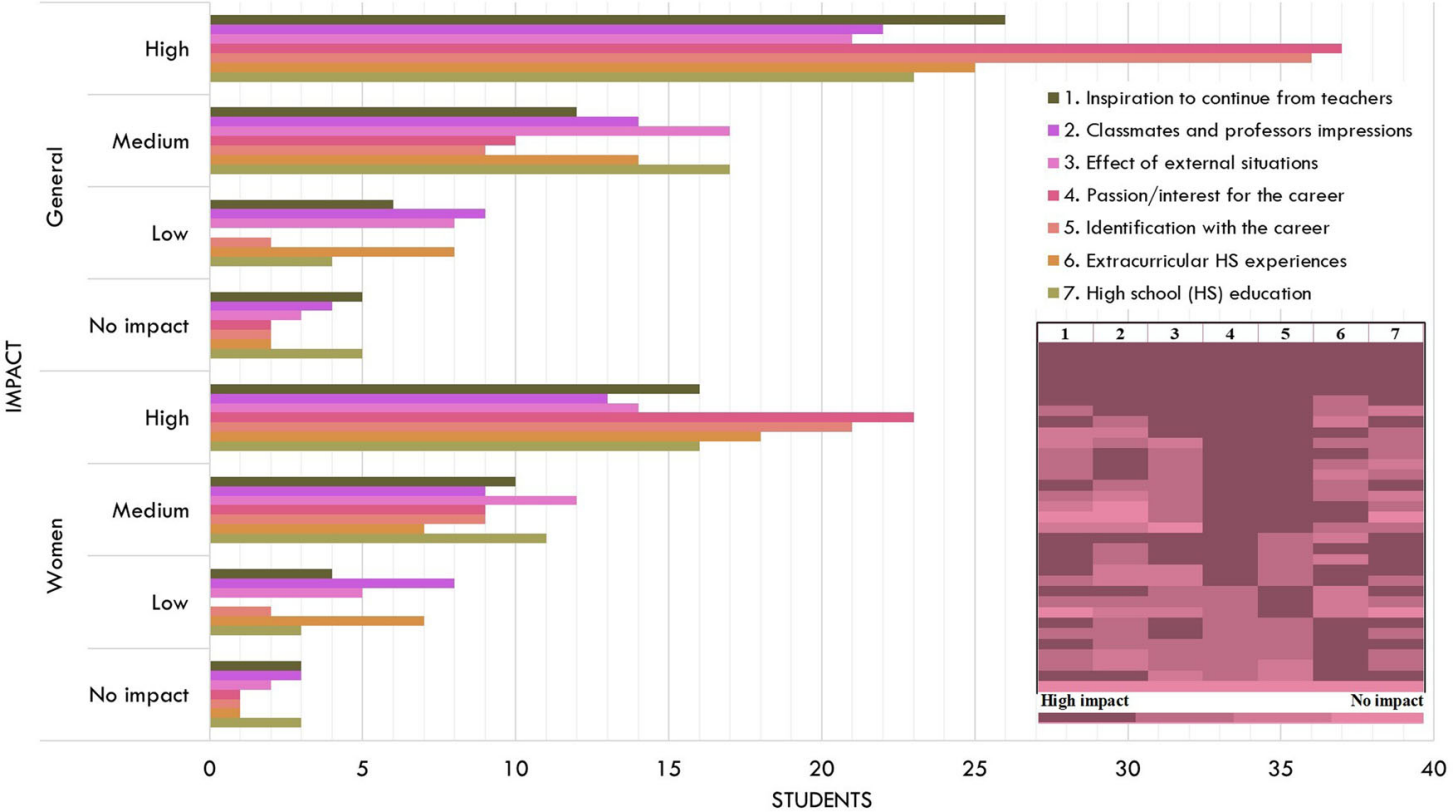
Impact perception on seven factors that may affect STEM career choice. The square insert below the legend is a color diagram showing the preferences of female students.

The last section of the survey asked students to choose an option according to their perception of the impact of some factors on their career choice (Fig. 6). As observed in Fig. 6, the students’ perceptions (regardless of gender) were that *passion, personal interest*, and the subjects (*identification*) influenced their career choice and significantly affected retention in a STEM career. However, some students chose all the options, considering they all had a high impact. High school extracurricular experiences also affected female students, as shown in the color chart. The effect of external situations had a medium impact, with factors 4 and 5 (passion and identification with the career) being the most accepted.

When the students were asked to write anything else they would like to add or recommend for someone thinking about or pursuing a career in STEM, students wrote:

- “We need to teach new students more about what they can do, what they can learn, and that it’s not as many say in terms of difficulty. You don’t have to be a genius to get into an engineering career; although it may not be easy for everyone, it’s worth not giving up”.

- “I would like to mention that, as women, we still perpetuate the idea that we do not belong to the STEM area. This only limits our participation in this discipline. For this reason, I consider it very important to continue implementing actions that promote and foster interest, regardless of gender.

-Be curious about your surroundings and observe what interests you the most. Try to have a solid foundation in physics, chemistry, math, and biology.

-Investigate what you will enroll in to see if you like spending much time in the laboratory.

-Think about it very well and talk to graduates of the race to find out what to expect.

In sum, considering the women interviews, the internal and external factors that affect decision-making and permanence in a STEM career, related to the SCCT theory, are shown in Table 6. Faculty may be considered a positive factor when transmitting the passion for knowledge, motivation, friendliness, support, solidarity, empathy, companionship, and mentoring students about the future, but harmful if not. Personal experiences of success in any STEM course, with stakeholders, graduated students, and available STEM information, increase their interest in these careers. Students’ self-perception corresponds to the characteristics and values of students that make them choose a STEM career. The most mentioned answers were creativity, altruism, perseverance, sustainability, curiosity, resilience, and persistence. A toxic competitive environment, excess of information in some classes, and Faculty were classified as external damaging factors.

**Table 6:** Internal and external factors affect decision-making, permanence, and reasons that cause the choice or not of a STEM career.

	Factors	Count
Internal	Self-perception	41
	Interests	17
	Personal experience	7
	Self-efficacy	5
External	Faculty	27
	Extracurricular courses	17
	Peers’ commitment	6
	Challenges/Stakeholders	4
	Family support and communication	2
	Multidisciplinary	1
	Curricular courses	2
	University environment	1
	Networking	1

Codification of women’s answers.

### Implications of the study

The data obtained show us that there is a significant gap in the percentage of female students in STEM careers. Much work must be done to reduce that difference and have fully inclusive academic programs. Analyzing the responses taken up to this point, internal factors influencing female students’ selection of STEM careers can be summarized into the *student’s interests and self-perception*. This comes from the beginning of their interaction with an academic environment. Additionally, the role of the family is fundamental. Some students mentioned that the career choice was agreed upon with their families. The external factors correspond with *extracurricular experiences*. Several responses indicated that the available workshops on robotics and biology gave them the necessary experience to know what STEM subjects they could find. STEM careers are much broader in scope than high school classes, and workshops can be a great tool to promote science pathways. Students could easily tackle projects and courses from the other STEM strands. Nonetheless, a second external factor, *Faculty*, has much to do with *young people’s perceptions* (internal factor). There are solid criticisms of the Faculty’s passion when teaching STEM subjects. Perhaps an analysis of the academic load of Faculty would be an essential point. An intermediate review of the opinion of students during a STEM course would be crucial.

These results go hand in hand with the theoretical framework of the SCCT, as indicated by other works. Hughes (2018) mentions that although gender bias still exists in HE, and these negative experiences must be studied as systemic and specific problems, the sense of belonging and identification with a STEM career is the strongest predictor of retention. This identification can develop during high school education or even before. Internal motivation and commitment are linked to persistence and resilience (Blackburn, 2017), and this will increase as adaptability and commitment increase in learning communities. Almukhambetova et al. (2021) highlight the effect of the role model on students’ beliefs, interests, goals, and actions that affect their performance. We have found that implementing experiences designed for student engagement represents a successful strategy for increasing student retention in STEM careers, particularly in minority groups such as women (Ortiz-Martínez et al., 2019).

We propose to create *mentoring groups for women in STEM careers*. Mentoring communities allow interaction between students and professionals in their areas of interest through experiential learning (Campos et al., 2022). Mentors create spaces to interact with students about their progress and sense of belonging. Through this interaction, collaborative networks can be made that guarantee the permanence of students, supported by motivation and follow-up, reducing anxiety. Additionally, we propose to create *digital platforms to have support networks for students*, such digital resources must have assigned tutors trained to give workshops that increase awareness about the personal and social problems that women face daily and that are sometimes considered taboo (since the self-perception to the economic aspect or unique situations such as mothers who want to obtain a professional degree, or even cases of harassment, sexual health, etc.). By measuring the success of these implementations, it will be possible to lay the foundations for long-term objectives that increase women’s sense of belonging in STEM areas by developing educational innovation practices in different courses and supporting the teaching staff to understand the diversity of the students. *Systematic Faculty training* is also required, with current issues, with an understanding of the characteristics of the students of this generation and considering the factors that affect the local, regional, and global environment, such as the COVID-19 pandemic.

Related works have suggested *access to family support* and an *increase of the sense of belonging*, through family communication hubs and buddy systems, especially during the first two years of study, and develop programs for families to give them advice on how to support students at a distance (Sosu and Pheunpha, 2019). Also, suggestions for student retention have been made based on the Attribution Theory of Achievement Motivation and Emotion (Weiner, 1985), such as the increase of *predictability and controllability* via well-structured courses, timely and constructive failure feedback, and articulated task expectations; and longitudinally monitor students perception of control over their academic outcomes to foster their long-term university success (Respondek et al., 2020).

### Study limitations

This work is limited to descriptive statistics. Future work will benefit from a more quantitative analysis of the results and strategies of in-depth coding and analysis of students’ responses. Even though this study has conclusions that are very close to the reality of the lower percentage of women in STEM careers compared to men in any university, the study was conducted in a private institution. This has an implicit bias because to access the university, although around 70% of the students have an academic scholarship, payment is required, and access is restricted. This may lead us to think that the interest in pursuing a STEM career may differ from other universities, including public ones. Although the general statistics of public universities may be like those analyzed here for a private university, the fact that the dropout rate of women who studied in public high schools was 9.97%, compared to the 7.14% dropout of women who studied in private high schools (UDH data) is noteworthy to mention. Another limitation is the small number of students surveyed. However, the number of surveys is justified by the current situation of returning to the classroom and the re-adaptation of students to the new reality. Though analyzing responses related to difficulties adapting to Faculty and peers, this situation was not exclusive to women. This may indicate that some concerns regarding the permanence of women in STEM are shared with men. In future studies, these situations should be studied more closely with the findings obtained for non-binary students.

## Conclusions

The percentage of women in STEM majors or the retention of female students in them is lower when compared to their male counterparts. Data from other studies confirm that it is a multifactorial phenomenon requiring immediate attention and actions for correction. The most significant value of this contribution is conducting a study in a controlled environment where rapid changes and actions can be implemented and results monitored in a controlled manner. Our research questions were successfully addressed. We detected internal and external factors and situations that affect decision-making, permanence, and reasons that cause the choice or not of a STEM career (1). Some were self-perception, interests and self-efficacy, faculty, extracurricular courses, peers and family commitment, environment, and networking. Also, based on our data, we made specific proposals that could favor retaining women already enrolled in STEM careers (2), such as remote mentoring programs, digital networking platforms, and systematic Faculty training. By implementing these proposals, monitoring compliance with indicators should be established to assess whether the percentage of women enrolled and retained in STEM careers increases.

The factors that we found in this work were consistent with the SCCT. First, the women’s interest in STEM careers was raised by personal experiences (extracurricular activities, family support, information access, and stakeholder interaction). Second, those students who choose a STEM career rely on self-efficacy (personal experiences of success in classes) to persevere. Third, once in the career, external factors such as Faculty, peers, and the university environment contribute to validating their goals or conduct them to fail. In this regard, proposals to increase women’s participation in STEM careers must be made to (1) increase students’ interest in STEM careers and (2) support and monitor the accomplishment of their goals, especially during the first and second years of their careers.

This work contributes to an institutional research project on lifelong education for women’s personal and professional well-being. The accompaniment proposal through a mentoring model has started, and the results will be presented in subsequent publications. Our data indicate that differences still exist in women’s self-perception and affinity for STEM careers. Changing the mentality is a difficult challenge, but if it starts from High School with extracurricular activities, STEM workshops, talks, or interactions with experts or professionals on STEM topics or on the academic and work benefits of being a female professional in a STEM area, we will have an excellent start to reverse the percentages. Every time more women could enter college (mainly to show independence; Mead, 2022), however, they are less likely to enter STEM majors and, once there, more likely to drop out (Vooren et al., 2022). Why does it have to be like this in all the cases studied? We do have to take action. We hope that our results will cause a change in the way of approaching the problem and allow us to follow up on those factors that negatively affect women in STEM careers. In any case, this work will help discuss this problem that we will have to reverse in the future to give women the same opportunities and benefits as men.

## Supplementary information

Supplementary Material

## Data Availability

The datasets used and/or analyzed during the current study are available from the corresponding author upon reasonable request.
