# Supplementary material for: Analysis of the retention of women in higher education STEM programs
Source: Humanit Soc Sci Commun. 2023 Mar 11;10(1):101. doi: 10.1057/s41599-023-01588-z (PMC10007666; doi:10.1057/s41599-023-01588-z)
Supplement: Supplementary file 1 — Supplementary Material [file 41599_2023_1588_MOESM1_ESM.docx]

**Analysis of the retention of women in Higher Education STEM programs**

Gabriela Ortiz-Martínez^1&^, Patricia Vázquez-Villegas^2&^, María Ileana Ruiz-Cantisani^1^, Mónica Delgado-Fabián^1^, Danna A. Conejo-Márquez^2^ and Jorge Membrillo-Hernández^1,2^*

^1^ School of Engineering and Sciences and ^2^Institute for the Future of Education, Tecnologico de Monterrey, Mexico

^&^These authors contributed equally to the manuscript and share the first authorship.

*Correspondence: jmembrillo@tec.mx

**Supplementary material**

**Survey: Factors that impact staying in a STEM program.**

**(Translated from Spanish)**

Disclosure: Thank you for participating in this research. The objective is to know the factors that impact the decision to choose and stay in a career. The results of this survey may be published for academic purposes.

**Section 1. General data**

4. Campus

5. Gender (Male, Female, Other)

3. Semester of admission to Tec de Monterrey

2. Do you agree to participate in a further research interview (voice recording)? Yes/No

6. During high school, did you take courses in (Physics, Mathematics, Chemistry, and Computing)?

7. During high school, did you participate in any activity, project, student group, or congress related to any ​​engineering, science, or technology area? Yes/No

8. If the answer was yes, describe the activity(ies) in which you participated (for example, robotics workshops, computer workshops, etc.).

9. Are you currently pursuing a career or avenue in Engineering, Science, or Technology? Yes/No

10. Write down the current program or avenue in which you are enrolled.

11. What identifies you with the career you are currently studying?

12. Is the program you are currently enrolled in the same one you started with? Yes/No

13. If not the same program/avenue, which one did you enroll in first?

14. Which of the following situations best describes you?

a) I have always been in an Engineering, Science, or Technology career

b) I changed my career to studying Engineering, Science, or Technology currently.

c) I changed from Engineering, Science, or Technology to another major (business, medicine, humanities)

**Section 2. If you are in a career or avenue of Engineering, Science, or Technology from the beginning at Tec de Monterrey.**

1. What other people’s attitudes have impacted you to stay in engineering, science, or technology studies?

2. What self-attitudes do you think have impacted you to stay in engineering, science, or technology studies?

3. What situations have impacted your stay in engineering, science, or technology studies?

4. What actions in your classes, by teachers and students, have impacted you, to stay or not in engineering, science, or technology studies?

**Section 3. If you changed your career or avenue from another to currently studying Engineering, Science or Technology.**

1. What other people’s attitudes have impacted you to change your studies to an engineering, science, or technology career?

2. What self-attitudes and values do you think have impacted you to change your studies to an engineering, science, or technology career?

3. What situations have impacted you to change your studies to an engineering, science, or technology career?

4. What actions in your classes, by teachers and students, have impacted you to change your studies to an engineering, science, or technology career?

**Section 4. If you changed from Engineering, Science or Technology to another major (business, medicine, humanities, etc.)**

1. What other people’s attitudes have impacted you in the change in your studies to a career other than engineering, science, or technology that you initially selected?

2. What self-attitudes and values do you think have impacted you in the change in your studies to a career other than engineering, science, or technology that you initially selected?

3. What situations have impacted you in the change in your studies to a career other than engineering, science, or technology that you initially selected?

4. What actions in your classes, by teachers and students, have impacted you in the change in your studies to a career other than engineering, science, or technology that you initially selected?

**Section 5. Closure**

1. After this reflection, what factors most affect your selection and permanence in a Science, Technology, Engineering, or Mathematics career?

a. High school education (courses in physics, chemistry, mathematics).

b. Previous high school experiences related to engineering, science, or technology.

c. I identify with the career I selected.

d. My passion/interest for the program I selected.

e. Situations that affected me to be in an engineering, science, or technology career.

f. My classmates and professors impress me to continue in my career.

g. Mentors/advisors/teachers who have inspired me to continue.

2. Write anything else you would like to add or recommend for someone who is considering studying a career in engineering, science, or technology.

Thank you very much for your participation and for helping us better understand how to support you in your decision-making process in a vocation.
